# Migrant women’s experiences of pregnancy, childbirth and maternity care in European countries: A systematic review

**DOI:** 10.1371/journal.pone.0228378

**Published:** 2020-02-11

**Authors:** Frankie Fair, Liselotte Raben, Helen Watson, Victoria Vivilaki, Maria van den Muijsenbergh, Hora Soltani, the ORAMMA team

**Affiliations:** 1 Department of Nursing and Midwifery, Sheffield Hallam University, Sheffield, England, United Kingdom; 2 Department of Primary and Community Care, Radboud University Medical Centre, Nijmegen, Netherlands; 3 Department of Midwifery, Faculty of Health and Caring Sciences, University of West Attica, Athens, Greece; 4 Pharos, Centre of Expertise on Health Disparities, Utrecht, Netherlands; Ben-Gurion University of the Negev Faculty of Health Sciences, ISRAEL

## Abstract

**Background:**

Across Europe there are increasing numbers of migrant women who are of childbearing age. Migrant women are at risk of poorer pregnancy outcomes. Models of maternity care need to be designed to meet the needs of all women in society to ensure equitable access to services and to address health inequalities.

**Objective:**

To provide up-to-date systematic evidence on migrant women’s experiences of pregnancy, childbirth and maternity care in their destination European country.

**Search strategy:**

CINAHL, MEDLINE, PubMed, PsycINFO and Scopus were searched for peer-reviewed articles published between 2007 and 2017.

**Selection criteria:**

Qualitative and mixed-methods studies with a relevant qualitative component were considered for inclusion if they explored any aspect of migrant women's experiences of maternity care in Europe.

**Data collection and analysis:**

Qualitative data were extracted and analysed using thematic synthesis.

**Results:**

The search identified 7472 articles, of which 51 were eligible and included. Studies were conducted in 14 European countries and focused on women described as migrants, refugees or asylum seekers. Four overarching themes emerged: ‘Finding the way—the experience of navigating the system in a new place’, ‘We don't understand each other’, ‘The way you treat me matters’, and ‘My needs go beyond being pregnant’.

**Conclusions:**

Migrant women need culturally-competent healthcare providers who provide equitable, high quality and trauma-informed maternity care, undergirded by interdisciplinary and cross-agency team-working and continuity of care. New models of maternity care are needed which go beyond clinical care and address migrant women's unique socioeconomic and psychosocial needs.

## Introduction

International migration continues to grow rapidly [1]. Between 2000 and 2017, the migrant population increased by 85 million, from 173 to 258 million [1]. In 2017, more than 90 million international migrants were residing in the World Health Organization (WHO) European region and more than half of these migrants were women, many of childbearing age [2]. There are no universally accepted definitions for a migrant at an international level [2] and this heterogeneous group includes individuals who vary by length of stay in a country, documentation and residency status, movement being voluntary or forced, and reasons for migration [2,3]. Health needs and outcomes in this heterogeneous group is a complex topic, as these are influenced by the interaction of the process of migration and exposure to risks and access to the determinants of health in the country of origin, during transit and in the destination country [2].

On average the fertility rate in the migration population is higher than the native population [4]. Among women living in the United Kingdom, birth data from 2015 show a total fertility rate (the average number of children a woman has in her lifetime) of 2.06 for non-UK born women versus 1.75 for UK born women [5]. Pregnancy is a period of increased vulnerability for migrant women [6,7]. There is a consistent trend for poorer pregnancy outcomes amongst migrant women [2] who are at greater risk of maternal and neonatal morbidity and mortality when compared to native born women [2,8–17]. This is a result of the complex interplay of multiple factors including substandard healthcare in the country of origin [2] and issues around accessing care and the quality of care in the new country [2,14,18]. Moreover, migration itself can have significant negative consequences for people's physical and mental health and their wellbeing due to migration-related social problems, like poor socio-economic status, discrimination and social exclusion, multiple losses, and the chronic stress caused by these [19–21]. It is often observed that migrants leaving their country of origin are healthier than comparable native populations. This phenomenon has been called the “healthy migrant effect” and is usually explained through the positive self-selection of immigrants and the positive selection, screening and discrimination applied by host countries [22]. But, although often healthy when arriving in the country, the health of migrants deteriorates over time, and in general, they rate themselves to have poorer health compared to the native population of their host countries [20].

Across the WHO European region there is consensus and commitment to ensure the availability, accessibility, affordability and quality of essential health services for migrants in transit and host environments [23]. Hence European countries have a common responsibility to tackle inequalities and provide high quality healthcare that meets the needs of childbearing migrant women. However across European Union (EU) member states, the services provided for migrants and how they are administered, financed and delivered differs between countries; with some providing care free of charge, some requiring health insurance and some available to those making national insurance contributions through a place of work [24].

A previous qualitative evidence synthesis [25] has explored both migrant women's care experiences and their perceived care needs for data published prior to June 2010. However, an updated review was deemed important with the acknowledgement that changing global, political and economic climates have led to increased migration into Europe [2,26]. This includes recent political unrest and conflict in many Middle Eastern and Sub-Saharan countries [26], the updated rights of free movement of citizens and their families within the European Economic Area laid down in a Directive in 2004 [27] and an increased recognition of the need to integrate the health needs of migrants and refugees into national health strategies [2]. This review therefore aimed to provide up-to-date systematic evidence on migrant women’s experiences of pregnancy, childbirth and maternity care in their destination country within Europe.

## Methods

A systematic search of five databases was undertaken to identify articles pertaining to migrant women's experiences of pregnancy and maternity care in their destination country. The following databases were searched; CINAHL, MEDLINE, PUBMED, PSYCHINFO and SCOPUS. Databases were searched from 2007 until the final search on 22/05/2017. The point of commencement was taken as 2007 due to the changing political landscape within the EU at that point, with the health of migrants being a focus of the EU president in 2007 [28]. The search strategy comprised of three facets, with terms relating to (i) migrant (ii) maternity and (iii) experience. The Boolean operators AND and OR were used alongside truncation operators and phrase-searching, and the search syntax was adapted for each database. The full search strategy, as applied in MEDLINE (EBSCO interface) is provided in S1 File. In addition to the electronic database search, the reference lists of eligible studies were examined to identify any other relevant studies and citation tracking was undertaken.

### Study selection and data extraction

Screening of the titles and abstracts against the inclusion and exclusion criteria in Table 1 was carried out by two researchers independently. This was followed by double-screening the full-text of potentially relevant sources. Any disagreements concerning eligibility were resolved through discussion between team members. Study characteristics and all qualitative data that related to women's experiences of any aspect of maternity care within the host country were extracted using a standardised form.

**Table 1:** Inclusion and exclusion criteria.

Inclusion Criteria	Exclusion criteria
• Qualitative or mixed-methods studies with a qualitative component • Peer reviewed articles • Exploring any aspect of migrant women's experiences of maternity care in the host country • Study undertaken in Europe • Published within the last 10 years (from 2007 onwards) • Studies focussed on women described as migrants, refugees or asylum seekers, including undocumented migrants • Where both first and second-generation migrants were included within a study, the study was included but where possible only the views of the first-generation migrant women were included • Where studies included both the experiences of migrant women and health care professionals, only the views of the migrant women were included • No language restrictions were put in place	• Internal migrants (eg rural to urban) • Migration status unclear (eg. studies of ethnic minorities women with no reference to migration status) • Non peer-reviewed articles eg commentaries, editorials, reports, books, protocols and theses/dissertations • Systematic reviews and reviews—however their references were systematically searched • Studies focussed solely on women's experiences of interventions

### Critical appraisal

Included articles were quality appraised using the qualitative National Institute for Health and Care Excellence (NICE) critique tool [29] (see S2 File) and 10% were appraised by a second reviewer to ensure consistency. A low-quality score (-) was assigned if either most criteria were not met, or it was judged that there were significant flaws in the study design. The article was classified as moderate quality (+) if most criteria were met and it was identified that there may be some flaws in the study resulting in a lack of rigor. A high-quality score (++) required that the majority of the appraisal criteria were met and the study was judged to be trustworthy and reliable and there was significant evidence of author reflexivity.

### Evidence synthesis

A thematic synthesis was undertaken involving 3 separate steps; i) line by line coding adding new codes to the 'bank' of codes as required, ii) organising codes into descriptive themes according to their similarities or differences and using new codes to capture the group of original codes, iii) generating analytical themes [30]. Coding was undertaken using NVivo and Atlas.ti packages. A total of 28% of the articles were double-coded, and development of the final analytic themes involved discussion with the whole research team to achieve consensus.

### Confidence in the findings

The confidence in the findings of this review was assessed independently by two reviewers using the Confidence in the Evidence from Reviews of Qualitative Research (CERQual) approach [31,32]. This assesses confidence in the evidence base in four components: (i) methodological limitations which evaluates any methodological concerns in the primary studies contributing to the review finding, (ii) relevance to the review question evaluates the applicability of primary study data to the context specified in the review question, (iii) coherence which evaluates the fit between the primary study's data and the review finding it contributes to and (iv) adequacy of the data which evaluates the richness and quantity of primary study data for each review finding [33]. An overall judgement for confidence in each review finding of 'high', 'moderate' or 'low' was determined based on evaluation of the four components.

## Results

**Fig 1:**
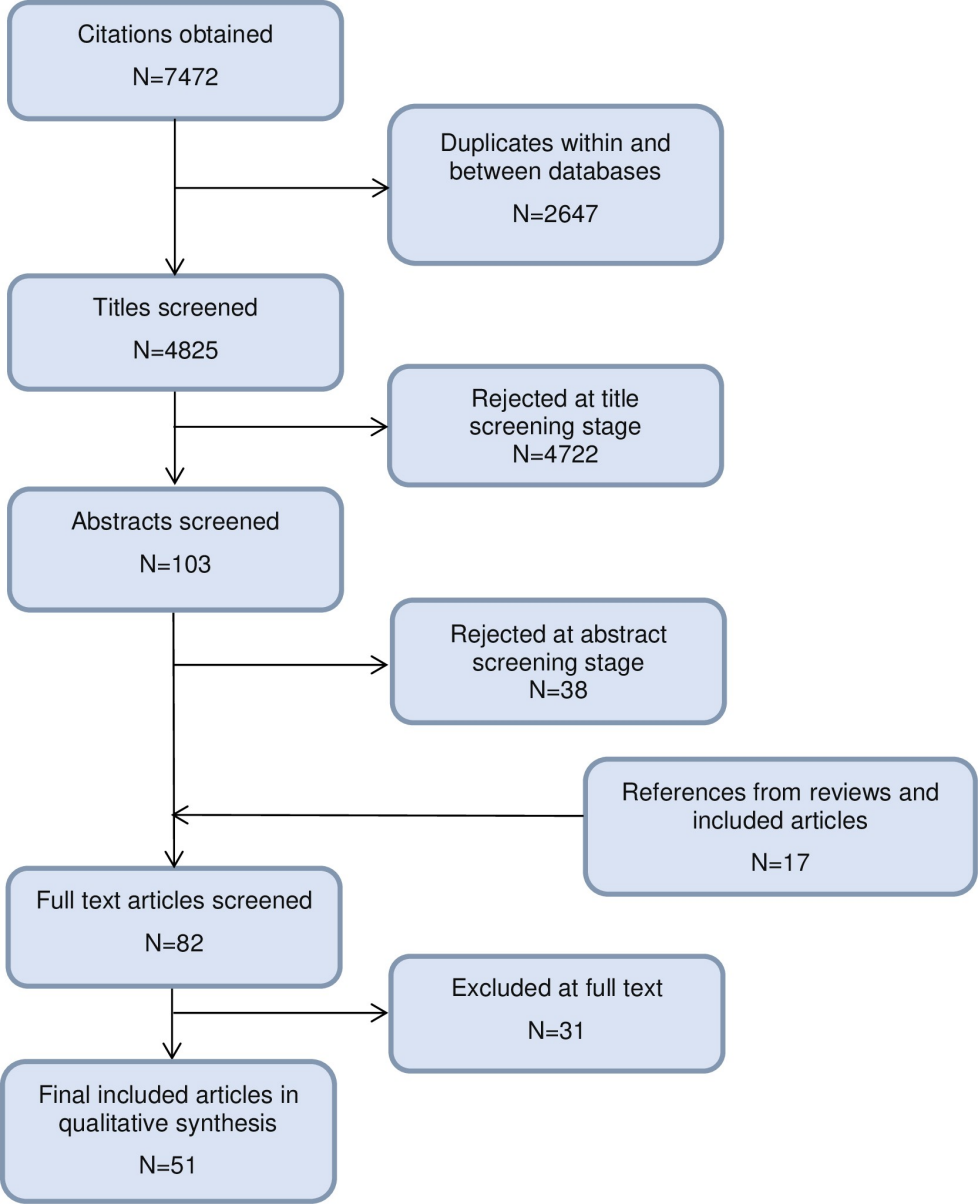
Flowchart of study selection.

A flow diagram of the study selection process can be seen in Fig 1. A total of 7472 citations were initially identified out of which 51 articles (47 studies) were included.

### Description of included studies

The characteristics of the included studies can be seen in Table 2 and the reasons for exclusion at abstract and full text can be found in S3 File. Of the 47 included studies, 43 exclusively used qualitative methodology and four adopted a mixed methods approach and reported relevant qualitative data [34–37]. Individual interviews were exclusively undertaken in 27 of the studies [8,38–63] and focus groups in five studies [64–68]. Multiple methods of data collection were used in fourteen studies [34–36,69–79] including eight which conducted both interviews and focus groups with different groups of women [69,71–75,77,79]. One study used a questionnaire which included relevant qualitative data [37]. Studies were undertaken in 14 European countries, ranged in size from four [70] to 193 [37] participants and included a total of 1330 migrant women, although one study did not specify the number of participants and could not be included in this number [34]. The majority of studies (n = 34) were published from 2012 onwards. A total of seven studies were rated as high quality [35,40,60,64,67,71,74], 22 were of moderate quality [38,39,41,43,45,46,48,53,55–57,61–63,65,70,73,75–79] and 18 of low quality [8,34,36,37,42,44,47,49–52,54,58,59,66,68,69,72].

**Table 2:** Characteristics of included studies.

**Qualitative studies**												
* studies marked with an asterix are taken as the primary report for that study												
**First author (year)**	**Study design**	**Setting (country research undertaken in)**	**Participants**				**Aim**	**Data collection**	**Data analysis**	**Outcomes**	**Comments**	**Quality score**
			**sample size**	**country of origin**	**age**	**parity**						
Almeida & Caldas (2013) [8]	Qualitative	Portugal	14	Brazil (n = 7) and Portugal (n = 7)	Not reported	Not reported	To investigate native Portuguese and immigrant women’s perceptions of maternity care.	Semi-structured interviews	Qualitative content analysis.	Brazilian women were dissatisfied with the quality of information provided by the health professionals, the communications skills of these professionals, and reported reduced access to medical specialties, especially in primary care	Only results from migrant women were used	-
*Almeida, Caldas et al (2014) Almeida, Casanova et al (2014) [38,80]	Qualitative	Portugal	31	African countries (11), Eastern European countries; (7), Brazil (7) and 6 Portugal	20–45 years	Not reported	To investigate native and immigrant women’s perceptions about quality and appropriateness of maternity care	Semi-structured interviews	Qualitative content	Misinformation about legal rights and inadequate clarification during medical appointments frequently interacted with social determinants, such as low social-economic status, unemployment, and poor living conditions, to result in lower perceived quality of healthcare.	Only results from migrants were extracted	+
Babatunde & Moreno-Leguizamon (2012) [64]	Qualitative	UK	17	Nigeria (11), Ghana (2), Kenya (1), Somalia (1), Sierra Leone (2)	16–45 years	Not reported	To establish cultural elements related to postnatal depression through women’s narratives.	Focus groups	Thematic analysis	Women who experienced postnatal depression did not perceive the signs as related to illness but as something else in their daily lives. Depression was not identified by health visitors, despite prolonged contact with the women.		++
Barona-Vilar et al (2013) [65]	Qualitative descriptive and exploratory study	Spain	26 immigrant women and 24 midwifes	Bolivia and Ecuador	20–35 years	1- >2 children	To explore the perceptions, attitudes and experiences of Ecuadorian and Bolivian women with regard to motherhood, pregnancy and their experiences of the health-care system.	Focus groups	Content analysis	Women reported that it was not necessary to go as soon and as frequently for health examinations during pregnancy as the midwives suggested. The main barriers identified to health-care services were linked to insecure or illegal employment status, inflexible appointment timetables for prenatal checkups and sometimes to ignorance about how public services worked	Only results from migrant women were used	+
Binder, Johnsdotter et al (2012) [39]	Qualitative. Hermeneutic	UK	54 immigrant women and 62 NHS maternal care providers	Sub-Saharan regions in Africa (Somalia, Ghana, Nigeria, Senegal, Eritrea).	18–48 years	1–10 children	To explore the influence of pre-migration socio-cultural factors on post-migration maternal care-seeking, and barriers between immigrant women and maternal care providers during the care encounter.	Semi-structured interviews	Constant comparison and triangulation with framework	Broken trust between women and maternal care providers may result in delays at the facility level, expressed as women’s choice for late-booking, non-adherence, or inappropriate decision-making, and as provider frustration resulting from the inability to impart optimal treatment.		+
Binder, Borné et al (2012) [69]	Qualitative	UK	50 immigrant women, 10 white British women and 62 obstetric care providers	Somalia (39) and Ghana (11) and UK (10)	18–48 years	1–10 children	To explore immigrant women's experiences of communication and conceptions of maternity care.	In depth- individual interviews and focus groups	Qualitative techniques using a framework of naturalistic enquiry.	Women encountered difficulties in health communication. Professionalism and competence were more important than meeting providers from one's own ethnic group. Interpreter use was limited by issues of quality, trust, and accessibility and has potential for improvement	Only results from migrant women were used	-
Bollini et al (2007) [66]	Qualitative	Switzerland	31 immigrant women and 9 native Swiss women	Turkey (14), Portugal (17)	Between <30 and >50 (not specified)	Between 1 and >2 children	To explore the issues of pregnancy and delivery in migrant women in their interaction with the Swiss healthcare system	Focus groups	Coding and the construction of themes	Migrant women face stressful situations, which may differ according to nationality and length of stay in the country. Main factors negatively affecting pregnancy were stress due to precarious living conditions, heavy work during pregnancy, inadequate communication with healthcare providers, and feelings of racism and discrimination in society	Only results from migrant women were used	-
Briscoe & Lavender (2009) [70]	Qualitative. Longitudinal exploratory multiple case study	UK	4	Afghanistan, Congo, Rwanda, Somalia.	19–36 years	1–3	To explore and synthesize female asylum seekers' and refugees' experience of maternity care	In-depth interviews. Photographs taken by the women. Field notes and observation.	Construction of themes	The women perceived ‘self’ as a response to social interaction. At times, ‘taken for granted’ communication in practice created a barrier to understanding for the women. Social policy related to seeking asylum, dispersal, housing and health affected the lives and maternity experiences of women		+
Byrskog et al (2016) [40]	An explorative, qualitative approach	Sweden	17	Somalia	18–45 years	Between 0 and >7 children	To explore how Somali-born women understand and relate to violence and wellbeing during their migration transition and their views on being questioned about violence in Swedish antenatal care	Individual semi-structured interviews	Thematic analysis.	A balancing act between keeping private life private and the new welfare system was identified, where the midwife’s questions about violence were met with hesitance. The midwife was, however, considered a resource for access to support services in the new society. A focus on pragmatic strategies to move on in life, rather than dwelling on potential experiences of violence and related traumas, was prominent. Social networks, spiritual faith and motherhood were crucial for regaining coherence in the aftermath of war. Dialogue and mutual adjustments were identified as strategies used to overcome power tensions in intimate relationships undergoing transition		++
Choudhry & Wallace (2012) [41]	Descriptive qualitative study	UK	20	South Asia. 11 born in UK, 9 outside UK.	Not reported	9 para 1, 9 para 2, 2 expecting first baby	To explore the influence of acculturation on breastfeeding practices of South Asian women.	Semi-structured interviews	Thematic analysis	5 themes - *‘Maa Kaa Dood’ (The mother’s milk)*; The most convenient method for me; Formula feeding as a way of fulfilling the baby’s demands; Breast isn’t always best – women’s experience of information and role conflict; Learning by observation – the formula feeding culture	Only results from migrant women were used	+
Coutinho et al (2014) [42]	Qualitative, exploratory, descriptive study	Portugal	82 (60 immigrant women and 22 native Portuguese women)	Brazil, Ukraine, China, Moldova, Russia, France, Span, India, Portugal and others	Not reported	Not reported	To identify the unmet expectations of the health system by Portuguese and immigrant women, during pregnancy, childbirth and postpartum.	Semi-structured interviews. Guidelines were used. Recorded.	Content analysis	Major emerging categories of unmet expectations referred to the accessibility, human resources, incentives to maternity care, physical and environmental conditions, and organization of the health system.	Only results from migrant women were used	-
Degni et al (2014) [67]	Qualitative	Finland	70	Somali women from Kenya (18), Mogadishu (32) and Hargeysa (20)	18–50 years	2–10 children	To explore immigrant Somali women’s experiences of reproductive and maternity health care services and their perceptions about the service providers	Focus groups.	Themes constructed	Participants were satisfied with the care they received in Finland. Despite their satisfaction, the health care providers’ social attitudes towards them were perceived as unfriendly, and communication as poor		++
Dempsey & Peeren (2016) [43]	Qualitative—grounded theory	Ireland	12	Eastern Europe	20–40 years	Varied, numbers not reported.	To explore migrant Eastern European women’s experience of pregnancy in Ireland	Semi-structured interviews	Construction of themes	Migrant women who experience pregnancy in their host country face multiple, multi-faceted challenges. Migrant Eastern European women may have particular struggles with transitioning to a less medicalised maternity healthcare system.		+
Essén et al (2011) [71]	Qualitative	UK	101 (39 Somali women and 62 obstetric care providers)	Somalia	18–48 years (Somali women)	1–10 children	To explore the attitudes of Somali women and their western obstetric care providers towards Caesarean section	In-depth semi-structured interviews and focus groups	Framework of naturalistic inquiry using the emic/etic model.	Somali women expressed fear and anxiety throughout the pregnancy and identified strategies to avoid caesarean section Avoiding or refusing caesarean was based on a rational choice to avoid death and coping with adverse outcome relied on fatalistic attitudes	Only results from migrant women were used	++
Feldman (2014) [44]	Qualitative	UK	20 women	14 different countries	Not reported	Not reported	To investigate the experiences of women who had been dispersed during pregnancy and of midwives involved in caring for these women	Individual interviews, face-to-face or telephone	Not specified	Dispersal interrupted women's access to maternity care. Women experienced practical barriers to accessing care and communication problems. Women experienced the postnatal period as emotional and stressful and had concerns about their living conditions.		-
Gardner et al (2014) [45]	Qualitative	UK	6	Nigeria and Ghana	22–26	1–3	To explore the lived experience of postnatal depression in West African mothers living in the UK.	Semi-structured interviews	Interpretive Phenomenological Analysis	West African mothers living in the UK experienced isolation and a lack of practical, emotional and professional support in the postnatal period.		+
Garnweidner et al (2013) [46]	Qualitative	Norway	17 (5 ethnic Norwegian and 12 immigrants)	Algeria, Albania, Pakistan, Thailand, Turkey, Russia, Sri Lanka, Somalia.	On average 28 years old	Not reported	To explore experiences with nutrition-related information during routine antenatal care among women of different ethnical backgrounds	Individual interviews	Interpretative phenomenological analysis	Participants reported that they were provided with little nutrition-related information. The information was perceived as presented in very general terms and focused on food safety. Weight management and the long-term prevention of diet-related chronic diseases had hardly been discussed. Women were confused about information given by the midwife which was incongruent with their original food culture. The participants were actively seeking for nutrition-related information	Only results from migrant women were used	+
Garnweidner et al (2017) [47]	An explorative qualitative approach	Norway	8 (5 immigrants and 3 ethnic Norwegian	Iraq, Turkey, Pakistan, Poland, Spain and Norway	Not reported	1–3 children	To investigate pregnant women's experiences of domestic violence and how this is addressed in antenatal care	Individual semi-structured interviews	Thematic analysis according to	Even though none of the participants were asked about domestic violence in antenatal care, they offered different suggestions on how and when midwives should talk about it.	Only results from migrant women were used	-
Gaudion & Allotey (2009) [72]	Qualitative	UK	43	Afghanistan, China, Eritrea, Ethiopia, Iraq, Iran, Sri Lanka, Somalia, Central and West Africa, Uganda, Zimbabwe and Russia	Many were teenagers who entered UK as unaccompanied asylum seeking children (otherwise NR)	Not reported	To describe refugee and asylum seeking women's experiences of pregnancy, childbirth and maternity services	Interviews and focus groups	Thematic analysis.	Women reported over stretched services, language and communication problems, issues around access and engagement, and the importance of cultural issues.	Teenagers were also included	-
Gitsels-van der Wal et al (2015) [48]	Qualitative	Netherlands	12	Morocco	20–36 years	0–3 children	To explore the preferences of pregnant Moroccan women regarding content of and approach to antenatal counselling for anomaly screening.	Interviews	Thematic analysis.	Women underlined the importance of accurate and detailed information about the tests procedures and the anomalies that could be detected and preferred counsellors to initiate discussions about moral topics and its relationship with the women's religious beliefs and values to facilitate an informed choice about whether or not to participate in the screening tests. Women preferred a counsellor who respects and treats them as an individual who has an Islamic background.		+
Glavin & Sæteren (2016) [49]	Qualitative	Norway	10	Somali	25–34	1–4 children	To explore Somali new mothers’ experiences of the Norwegian maternity health care system.	Semi-structured interviews	Content analysis	Findings highlighted inadequate integration into Norwegian society, the need for and fear of a caesarean delivery, issues of family support around the postpartum period and support from health services	Norway public health services cover all women and children	-
Hanley (2007) [68]	Qualitative	UK	10	Bangladeshi	16–24	1–4	To explore Bangladeshi mothers' interpretations of postnatal depression and its effect on the wellbeing on the mother, family and community.	Focus groups	Thematic analysis	When mothers experienced emotional issues they sought the support of their family, friends and religious leaders, and, although familiar with some primary care services, they were not always their first point of contact		-
Hjelm et al (2007) [50]	Qualitative	Sweden	27	Middle East (14) Sweden (13)	Mean age = 35	2 nulliparous 12 parous	To explore patients’ evaluation of a specialised gestational diabetes clinic	Semi-structured individual interviews	Content analysis	The healthcare model was perceived as functioning well. Women from the Middle East felt cared for, had been given the necessary information and claimed to follow advice. Adequate information reduced respondents’ anxiety and increased their control over the situation	Only results from migrant women were used	-
Hufton & Raven (2016) [73]	Qualitative	UK	35 (30 immigrant mothers and 5 maternal HCPs)	From 19 countries	Not reported	0–8 children	To explore infant feeding practices of immigrant mothers.	Semi-structured interviews and focus groups	Framework approach	Overall mothers were dissatisfied with their infant feeding outcomes. Mothers who were positive to human immunodeficiency virus followed the UK guidelines but struggled with guilt of not being able to breastfeed. All mothers unable to exclusively breastfeed experienced a sense of loss. Lack of wider support services coupled with complex lifestyles appeared to create challenges in providing infant feeding support	Only results from migrant women were used	+
Iliadi (2008) [51]	Qualitative	Greece	26	Iraq, Iran, Sudan, Lebanon, Syria, Afghanistan, Armenia, Turkey, Albania, Serbia, Zaire	Not reported	Primigravid (11), Multparous (15)	To examine whether refugee women, receive antenatal care and to explore possible factors that may influence their attitude towards maternity care	Semi-structured interviews	Latent content analysis	Analysis showed that refugee women enter antenatal care in the first trimester of their pregnancies, but they may miss from one to many appointments due to the language and financial barrier, the unfamiliarity with the national health system, and the women’s view of pregnancy as a natural event		-
Jonkers et al (2011) [52]	Qualitative—grounded theory	Netherlands	40 immigrant women (and 10 Dutch women) with severe maternal morbidity	Morocco Turkey, Suriname’ Dutch Caribbean Eastern Europe Middle East, Asian and sub- Saharan Africa	Not reported	Not reported	To investigate ethnicity-related factors contributing to sub-maternity care and the effects on severe maternal morbidity among immigrant women Netherlands	In-depth interviews	Thematic analysis	Women unaware of potential pregnancy complications and felt that HCP paid insufficient attention to pregnancy complications.	Only results from migrant women were used. It was not possible in this study to separate 1^st^/2^nd^ generation migrants	-
Lephard & Haith-Cooper (2016) [53]	Qualitative interpretive, in line with hermeneutic phenomenology	UK	6	Sub-Sahara Africa (4), Eastern Europe (2)	Over 18 otherwise not recorded	5 primigravid, 1 had 1 previous child	To understand the experiences of women seeking asylum while accessing local maternity services	Semi-structure interviews.	Thematic analysis	Women experienced pre-booking challenges, inappropriate accommodation, dispersal, being alone and not being listened to		+
Leung (2017) [54]	Qualitative	UK	10	China	Average age 36	8 primigravid, 2 mulitiparous	To explore how cultural beliefs influence postpartum dietary choices and infant feeding practices.	Semi-structured interviews	Not reported	Women felt midwives were unaware of their cultural practices when offering postnatal dietary advice		-
Lundberg & Gerezgiher (2008) [55]	Qualitative—ethnography.	Sweden	15	Eritrea	31–45 years	3 to 5 children	To explore Eritrean immigrant women’s experiences of female genital mutilation during pregnancy, birth and postpartum.	Semi-structured interviews	Thematic analysis	Women reported fear and anxiety, extreme pain and long-term complications and health-care professionals’ knowledge of circumcision		+
Ny et al (2007) [74]	Qualitative	Sweden	13	Turkey, Syria, Iraq and Lebanon	23–41	1–6 children	To describe Middle Eastern mothers' experiences of the maternal health care services in Sweden and the involvement of their male partner.	Focus group discussions and individual interviews.	Content analysis	Women developed trust in the midwife based on the knowledge and the empathy the midwife imparted, and did not feel that the midwife's understanding of their native language or culture was vital to develop a good relationship		++
Petruschke et al (2016) [56]	Qualitative exploratory	Germany	19 Turkish origin (11 German origin)	Turkey	21–41 years	42% nulliparous	To identify possible differences in the Turkish and German women’s attitudes towards epidural analgesia.	Semi-structured interviews	Content analysis	Turkish women ascribe meaning to labour pain and reject epidural for fear of long-term complications and because they don’t view epidural delivery as natural		+
Ranji et al (2012) [57]	Exploratory, qualitative	Sweden	9	Iran (5), Afghanistan (4)	21–39 years	2 nulliparous, 7 had one child.	To describe immigrant parents’ experiences of ultrasound examination in the second trimester of pregnancy	In depth interviews	Content analysis	Parents were impressed by the quality of their communication with the care-givers, found the process to be well organised and did not experience discrimination on the basis of being an immigrant		+
Robertson (2015) [75]	Intersectional approach	Sweden	25	17 countries	21–50+	Not reported	To analyse women’s reflections on how their migration and resettlement influenced their health and healthcare needs during childbearing.	Focus groups and semi-structured individual interviews	Content analysis	The hardships of migration, resettlement, and constraints in the daily life made women feel tense and disembodied. Being treated as a stranger and rejected in healthcare encounters was devaluing and discriminating. Women felt stronger and had fewer complications during pregnancy and labour when they had a confident, caring relationship with caregivers/midwives.	Interviews were a long time post delivery for some participants	+
Sauvegrain et al (2017) [76]	Qualitative	France	33	Sub Saharan Africa (16) France (17)	21–44	P1 = 12 P2 = 13 P3 = 3 P4 = 3 P6 = 2	To analyse whether the prenatal care trajectories among women with hypertensive disorders during pregnancy differed between immigrant and native women	Semi-structured interviews	Identification of themes	Some evidence of differential care.	Only results from migrant women were used	+
Strauss et al (2009) [58]	Ethnography	UK	8	Somalia	23–57	Not specified	To examine cultural and social aspects of childbirth and how they intersect with the needs and experiences of Somali women in the UK.	In-depth narrative interviews	Thematic analysis	Concerns raised around: the mismanagement of care for women who have been circumcised, aspects of communication, continuity of care and attitudes of health professionals		-
Szafranska & Gallagher (2013) [59]	Descriptive qualitative approach	Ireland	6	Poland,	Not reported	Not reported	To explore the factors that influence Polish women's decisions to initiate and continue breastfeeding in Ireland	Unstructured face-to face interviews.	Identification of themes	Professional and family support are key to successful BF		-
Tobin et al (2014) [60]	Qualitative Dramatisitic pentad	Ireland	22	9 different countries	18–40	9 primiparous, 13 multiparous	To gain insight into women's experiences of childbirth in Ireland while seeking asylum	In depth unstructured interviews	Narrative analysis	Women experienced a lack of connection, communication and culturally competent care		++
Topa et al (2017) [61]	Qualitative—critical feminist exploratory design with hermeneutic approach.	Portugal	10	Ukraine	28–49	6 x para 1, 4 x para 2	To investigate migrant women’s perceptions of the quality and appropriateness of maternity care received in public health services	Semi-structured interviews	Thematic analysis.	Women feel misinformed about their legal rights and free access to maternal health services. They were dissatisfied with the quality of information provided by HCP and their communication skills. They felt that their access to medical specialties was limited.		+
Treisman et al (2014) [62]	Qualitative	UK	12	Africa	23–41 years	Not reported	To investigate how UK-based African women perceive, make sense of, and manage a diagnosis of HIV during pregnancy, and after delivery	Semi-structured interview	Interpretive phenomenological analysis (IPA).	Receiving an HIV diagnosis challenged the normalcy and joy of becoming a mother. Women experienced stigma and breaches of confidentiality from HCP. Women found their inability to breastfeed most distressing as this was central to their cultural identity as mothers.		+
Viken et al (2015) [63]	Qualitative exploratory, descriptive design with hermeneutic approach	Norway	17	South America, Europe, Middle East, Africa, Asia	20–38	1–8 children	To explore the maternal health coping strategies of migrant women in Norway	Semi-structured interviews	Qualitative content analysis.	There were both good and bad experiences of care from HCPs during pregnancy and childbirth. Culture influenced the women's views of health and disease.		+
Wandal et al (2016) [77]	Qualitative	Norway	38 (16	Somalia	21–40 years	Majority multiparous	To explore infant feeding practices among Somali-born mothers in Norway, and the ways in which they navigate among different information sources	Semi-structured interviews and focus groups.	Development of categories	The mothers experienced challenges of dealing with conflicting recommendations and expectations regarding infant feeding. They navigated among different sources of information, taking into consideration traditional values, experiences and habits from living in Norway, and research-based knowledge.		+
*Wikberg et al (2012) Wikberg et al (2014) [78,81]	Ethnography	Finland	17	Australia(1), Bosnia (3), Burma (1), Colombia(1) Estonia (3), Hungary (1) India (1), Iraq (2) Russia (1), Thailand(1), Uganda (1), and Vietnam (1)	19–36 years	9x para 1, 4x para 2, 3x para 3, 1x para 4	To explore immigrant mothers' experiences of maternity care	Interviews, observations and field notes.	Focussed ethnographic analysis.	There were differences between the women's expectations and their maternity care experience. Caring was related to the changing culture. Finnish maternity care traditions were sometimes imposed on the immigrant new mothers. Female nurse was seen as a professional friend, and the conflicts encountered were resolved.		+
Yeasmin & Regmi (2013) [79]	Qualitative	UK	26	Bangladesh	20–44 years old	Most had more than 1 baby	To examine the food habits and beliefs of pregnant British Bangladeshi women	Focus groups and in depth semi-structured interviews	Identification of themes	Culture influence women's perceptions of 'good' and 'bad' food and their food habits during pregnancy.		+

**Mixed-method studies**												
* studies marked with an asterix are taken as the primary report for that study												
**First author (year)**	**Study design**	**Setting (country research undertaken in)**	**Participants**				**Aim**	**Data collection**	**Data analysis**	**Outcomes**	**Comments**	**Quality score**
			**sample size**	**country of origin**	**age**	**parity**						
Baken et al (2007) [34]	Mixed methods	Italy,	103	Natives and immigrants, no further details specified	Not reported	Not reported	To investigate immigrant's access to maternity health services	Quantitative research alongside Focus groups	Not reported	Migrant women faced several obstacles to accessing care including communication problems, limited knowledge of the health system in the new country, logistical barriers, limited family support and social inequalities	Only qualitative data extracted	-
*Phillimore (2015), Phillimore (2016) and Newall et al (2012) [35,82,83]	Mixed methods study	UK	95	28 different countries	Majority under 30	4 nulliparous 43 had 1 child, 27 had 2 children, 6 had 3 children and 2 had 4 children	To explore the reasons new migrant women book late for antenatal care and do not attend follow-up appointments	82 semi-structured questionnaires and 13 case studies using in-depth interviews	Thematic analysis of qualitative data and descriptive statistics for quantitative data	Structural, legal and institutional barriers prevent access to care. This includes language and communication, cultural health capital and discrimination, power and control, structural inequalities and social networks	Only qualitative data extracted	++
Schoevers et al (2010) [36]	Exploratory mixed method study	The Netherlands	100	32 different countries	Mean age of 36.4	Not reported	To explore the reproductive health problems of illegal female immigrants and obstacles they experience to seeking help.	Semi-structured interviews,	Identification of themes in qualitative data, and use of descriptive statistics for quantitative data	Obstacles to accessing reproductive health facilities include lack of information about services, financial problems, sexual and physical violence and fear of deportation	Only qualitative data extracted	-
Veleminsky et al (2014) [37]	Questionnaire with open and closed questions	Czech Republic	193	Vietnam (65), Mongolia (35) and Ukraine (93)	20–46	46.8% 1 child 37.1% 2 children,	To explore immigrant women's experiences of pregnancy and perinatal care	Semi-structured interview	Not reported	Failures in care included poor communication especially with a language barrier, limited possibilities for family to be involved in delivery and unsuitable HCP behaviours	Only qualitative data extracted	-

(green) ++ article judged to be of high quality as majority of NICE appraisal tool [29] criteria met. Study judged to be reliable and trustworthy, with evidence of author reflexivity

(yellow) + article judged to be of moderate quality as most criteria met in NICE appraisal tool, Study however deemed to lack rigor due to some flaws in study design

(red) - article judged to be of low quality as most criteria within the NICE critical appraisal tool not met

### Data synthesis

Four overarching analytic themes emerged from the literature.

#### Finding the way—navigating the system in a new place

*Weighing it up*. Before accessing maternity care women considered the value [35,51,52,60,81,82], and necessity [65] of care. They also weighed up the financial costs of accessing care [37,49,61], and the consequences of accessing care, particularly when they had a lack of trust in healthcare providers (HCPs) [39,75], previous poor experiences with HCPs [38], or were fearful that their visibility in maternity services could result in deportation [35,36,66,82].

*“I had my first daughter when I was illegal*, *it has been a terrible experience even though my sister helped me*, *I was always fearing that someone would knock at the door and would send us back to Portugal… Even when I had contractions I was afraid to go to the hospital fearing to be sent back to Portugal*.*"* (Bollini et al 2007, pp.82) [66]

*Finding the way in and through the system*. For some migrant women who wanted to access care, there were difficulties in finding the way into the system. The system was unfamiliar and different to that of their country of origin and the women were often unaware of their rights and entitlement to care [34,36,42,53,61,65,72,78,82,83]. There was a lack of information about the services that were available and if the services were free [36,53,61,82]. Some women faced difficulties in being accepted for registration for primary healthcare services [36,53,82], were refused entry to healthcare facilities [75], and struggled to provide the required documentation or insurance that were prerequisites for care [66,80]. Having friends and relatives who had already settled in the new country and could speak the local language helped migrant women find the way into the system, along with NGOs who provided information about entitlement and available services [36,51]. Women being held in detention centres were isolated from these sources of help and reported that the way into the system was blocked by detention centre staff who refused or delayed their access to care [35,53].

*"The Home Office put me in detention centre so I could not attend my appointments*. *There were no maternity services there for me for the 2 months I was there*. *I was offered appointments but they were cancelled at short notice without anyone telling me why*.*"* (Phillimore 2015, pp.576) [35]

Costs related to transportation and payment for care were identified as factors influencing ongoing access to care [34,44,53,61,83]. Those who received free care identified that this enabled them to access care, which was often in contrast to the situation in their country of origin [37,49,67,81]. Flexibility in the system in relation to the timing and location of appointments influenced access [61,65,70]. Inflexibility in the system, such as the rigid use of telephone booking systems for appointments were an ongoing barrier that women faced when trying to navigate the system in a new language [34,75,82].

*“I get so nervous to communicate through the telephone*, *is so difficult* … *instead I go there to get an appointment but they tell me I have to phone* …*Why*?*”* (Robertson 2015, pp.62) [75]

#### We don't understand each other

Women highlighted that information, advice and the opportunity to discuss their health and the health of their unborn child with a HCP was extremely important to them [63,74,78]. However, they identified a range of issues related to communication and understanding which are discussed in the sub-themes; Overcoming language barriers, Unmet information needs and Different expectations of care.

*Overcoming language barriers*. Women faced significant language barriers in the new country and felt that their language difficulties made them problem patients [69], that impacted on their relationship with their HCPs [37,53,66,78]. Even when women could proficiently manage everyday situations, they still often lacked the vocabulary to cope with medical terminology [53,58,70,75].

*"I asked them*, *“[Can] we cancel the meeting until we get an interpreter… I didn’t understand you and you didn’t understand me*.*” She said*, *“No*, *it’s OK*, *we can go on—you understand English*.*”’* (Lephard & Haith-Cooper 2016, pp. 134) [53]

Failure to use professional interpreters was a barrier to receiving satisfactory care [38,44,58,60,69,83], hindered accurate information sharing and led to frequent misinterpretation [52,70,81] and a lack of understanding of procedures women were asked to give consent for [35,52,60].

*“They [midwives] communicated by sign language and I was never sure I had understood properly*.*”* (Briscoe & Lavender 2009, pp.20) [707]

However, the use of professional interpreters was met with caution when discussing intimate or difficult matters [47,69,74,82] or when women had come from areas of persecution leaving them suspicious of everyone [75]. When women's partners were asked to interpret during care encounters some women felt vulnerable [35,82,83] and embarrassed [51,74] and felt that their partners were reluctant to reveal their own poor understanding [52,70,74].

*“If I could have someone who is not my husband it could make a big difference because throughout my pregnancy I did not say anything about my needs or problems*. *My husband was saying everything*.*”* (Phillimore 2015 pp.576) [35]

*Unmet information needs*. Women identified a lack of information around pregnancy, childbirth or the postpartum period, and a lack of information that was available in an accessible language or format [8,35,37,46–50,52,58,64,66,70–72,75–79,81–83]. Professional advice often conflicted with cultural and family advice [41,46,49,54,63,77–79] and this left women feeling insecure about which actions to take [46,63,77].

*"I did not give water*, *and I was criticized by my family and relatives*. *They told me*: *He is a human being*, *he gets thirsty and that milk does not quench thirst*… *while the health clinic said*: *no*, *he does not need water"* (Wandal et al 2016, pp.4) [77]

Women also identified that their care and safety were adversely affected when they did not disclose important information to HCPs, as did not want to be a nuisance or failed to understand the importance of their health history or potential seriousness of their current or previous symptoms [52,76].

*"I thought*: *it is a holiday*, *I do not want to be a problem for someone*. *I will try to go Monday or Tuesday after the holidays*. *But I think now*: *why did I wait* ? *Why didn't I phone immediately* ?*"* (Jonkers et al 2011, pp.149) [52]

*Different expectations of care*. Some women reported being fearful of being treated poorly in the new country when their expectation of maternity care was based on poor experiences in their country of origin [60,61].

*"I was so scared of them (the midwives)… I thought they would beat me…if I scream or if I cry*. *So in labour I don't speak*, *so that I don't upset them*.*"* (Tobin et al 2014, pp.836) [60]

Procedures which were familiar to practitioners were not always familiar to women coming from other care systems [8,70], and this caused women to feel fearful [60,82] and to lack trust in the information provided by HCPs [39].

*“They were putting all those funny cords around me which were so tight*, *so irritating*, *I didn't know what those were*, *I never had seen them before*. *It's like going to another planet and you are seeing all these things which are happening to you and you can't ask anything*.*”* (Tobin et al 2014, pp.836) [60]

Women's cultural backgrounds influenced some of their preferences [39,56,60,71] and beliefs about procedures [49,55,67,70,71,81] and the way they wanted to discuss these [56,74]. Experiences in their country of origin influenced their expectation of the need for medical surveillance and interventions during pregnancy and childbirth [8,42,43,63,80,81].

*"According to our religion*, *we Somali women*, *we don’t think that giving birth by caesarean section is a good thing and that a woman should give birth by vagina and not by opening her stomach to take the baby out*. *Somali women’s general belief is that caesarean birth is not a real way of a woman to give birth*. *And how many times doctors will cut her stomach if she has to deliver many times in her life*?*"* (Degni et al 2014, pp.357) [67]

*“I found it extremely friendly but very low in real medicine*? *It’s all midwife based*, *no exams*, *which is very strange for me”*. *(*Dempsey & Peeren 2016, pp.377) [43]

#### The way you treat me matters

*Impact of poor care*. The HCPs attitude was an important factor in how migrant women perceived the quality of care. Some women found HCPs to be unfriendly [67,74] and disrespectful [63,81], failing to respond to their concerns in a caring matter, ignoring them [74,75] and not taking their complaints seriously [49,52,66,74,75]. This made women doubt their own capabilities [75]. Unsatisfactory interactions with HCPs often led to a lack of connection and poor relationships with HCPs which resulted in women feeling isolated and fearful of being mistreated [60].

*"Really they should have asked in a friendly way if we needed help*…*it was a very unpleasant experience*, *I felt like an idiot*, *as totally incompetent*.*”* (Robertson, 2015, pp.63) [75]

When encountering the healthcare system, migrant women expressed a sense of being seen and treated differently [37,50,53,75,76]. Many women felt that their customs and culture were not understood by those caring for them [35,37,45,54,55,64,67,76,78,83]. Prejudice and stereotyping by HCPs [8,35,37,57,58,66,75,77,78] led to assumptions based on women's perceived cultural backgrounds and left them feeling that their needs were overlooked [35,52,53]. In contrast some HCPs were noted to overly focus on cultural and psychosocial factors when assessing patient’s symptoms, and therefore overlook potentially serious medical conditions [50,67].

*“I think that people that work in the health care settings … the doctors*, *the nurses*, *the midwives and even cleaners need education in different cultures*. *They need to understand that patients from different cultures and race are not inferiors and not* …*monsters*.*”* (Degni et al 2014, pp.360) [67]

Migrant women highlighted several other factors which resulted in inadequate and ineffective care including; long waiting times for appointments [61,80], the perceived busyness of HCPs which prevented women sharing their anxieties and concerns [70,81,82], inadequate knowledge of legislation by administrative staff [80], not being involved in decision-making [80], and limited access to specialist care [80].

*Importance of good care*. Women stressed the importance of good quality care and reported several examples from their experiences. They valued HCPs who were encouraging and reassuring [50,60,77], supportive [43,46,50,70,75] good listeners [50,71] and good information-providers [50,57,74]. Moreover, they wanted to be cared for by HCPs who had a respectful attitude [43,48,62,74], made them feel emotionally safe [43] and would take their concerns seriously [75]. Women also appreciated HCPs who demonstrated cultural sensitivity, although this did not necessarily require an in-depth knowledge of individual customs and traditions [48,78].

*‘You know when I talk about myself I feel good about it because I know there’s someone who’s listening and understanding which makes me feel better*.*’* (Briscoe & Lavender 2009, pp.20) [70]

Good care encompassed a trusting relationship between women and HCPs, which empowered women to feel confident and prepared for childbirth [63,75,78], even overcoming a lack of social networks or support [75].

“*When one feels well-treated and cared for*, *one never forgets it*…*especially when you feel lonely and vulnerable with a lot of need of support*…*it is worth so much*.*”* (Robertson 2015, pp.63) [75]

Continuity of care was seen as an important factor in establishing these trusting relationships [51,58,63,75,78,81]. Individualised care, with friendly, unhurried HCPs encouraged women to attend for maternity care and positively influenced their sense of well-being [37,74,81]. Fragmented care given by different midwives negatively influenced the effectiveness of care and the women’s confidence to attend appointments [82].

"*For example*, *when I was struck by panic again*, *I went to the delivery ward*, *and there was the same midwife*, *and (she) immediately knew me*. *Yes*, *she remembered the name and that it was the first pregnancy*, *it was nice*.. .. *It felt like she was a relative*.*"* (Wikberg et al 2012, pp.644) [78]

Women also identified that good care required facilities that were hygienic [37,74] and promoted privacy [81] and informed choice [74,78].

#### My needs go beyond being pregnant

Many migrant women presented to their HCPs and to the researchers in the primary studies with needs that were outside the ordinary remit of maternity healthcare provision and beyond the issue of their pregnancy. Preoccupation with these other needs impacted on their time and ability to focus on the pregnancy [35,36,62].

*"I was so busy to survive*, *to find food*, *and shelter*. *I simply did not think of antenatal checks at all*.*"* (Schoevers et al 2010, pp.260) [36]

*Financial difficulties and poor living conditions*. Financial pressures were identified by many migrant women which led to difficulties covering basic living costs [35,82,83], transport to appointments [35,53,72,82,83] and costs of essential care [51]. This was exacerbated by not being allowed to work in the host country [35,66,70,82] or difficultly securing a job [49,63,74,75]. Although some women encountered actual or feared employment insecurity [35,61,65,66,82] and exploitation [66], others appreciated the protection of national employment laws [81].

*“worst aspect I think during pregnancy he want to dismiss me […] but could not*, *could not because I had my rights*, *[…] but he fired me soon after the birth of my daughter” (*Topa et al 2017 pp.115) [61]

Concerns over living conditions were also common [44,52,53,62,66,70,73,83] and included; living in temporary [70] or shared accommodation [44,53], poor housing conditions [44,70] and the impact of dispersal [35,44,53,70,73,82], whereby women were moved by migration authorities to new, unknown areas within the host country. This increased women’s feelings of stress [44] and powerlessness [70].

*“They give me a [hotel] room… [It was] very small*, *it was smelling of cigarettes*. *The duvet was very dirty*. *The bed… the walls… everything was very dirty*.*”* (Lephard & Haith-Cooper 2016, pp.132) [53]

*“They were saying they’re taking me to Birmingham*. *I had no one in Birmingham*. *I don’t know anyone at all in Birmingham*. *I was like Oh God*, *where are they taking me*?*”* (Briscoe & Lavendar 2009, pp.21) [70]

*The burden of traumatic experiences*. Many childbearing women had experienced trauma or persecution prior to or during migration [45,52,60–63,75], and the resulting stress often became evident as pain and illness in their body [75]. These experiences left women with a lost or negative sense of identity [45,58,70] and being unwilling to trust their interpretations of their bodily symptoms [75].

*‘‘People were killed; I survived*, *because they thought I was dead*, *you can see the scars on my face*, *where the bullets entered my face* … *They did what they wanted with us*, *beating us*, *having rape parties"* (Treisman et al 2014, pp.150) [62]

*Social support and relationship issues*. Childbearing women who had family present in their destination country appreciated their assistance with domestic tasks [49,68,79] and their guidance [49,74,79,81], and support [56,59,71]. However, many migrant childbearing women lacked this social support and this left them feeling lonely [45,51,53,60,63,64,73,78,83], isolated [35,44,45,47,49,58,60,70,74,78,79], hopeless [51] and deeply distressed [37,60,70,74]. Women were particularly aware of the lack of support from their own mothers [45,53,60,74,78,81] and highlighted that being able to contact family members was important [63]. Without family support women were worried about having no one to ask for advice [74,78,81], found raising children more difficult [74,77,81] and felt that the changes in societal roles [61,75] and lack of other social support [40] caused tension in the relationship with their partners [75].

*“This was my first baby*, *I was afraid and also I don’t have family here*… *and was crying all the time and very lonely*.*” (*Babatunde & Moreno-Leguizamon 2012, pp.5) [64]

Women who experienced domestic violence were restricted from talking about this as it was often not acceptable within their culture [47] and they were not always aware that violence was forbidden in the destination country [47]. Where the woman experiencing abuse was also dependent upon the partners’ family for communication with HCPs it left her unable to talk openly about her circumstances or to report pregnancy problems [35]. Although the midwife was seen as a resource to signpost to domestic violence support services by some [40], others were unsure if a midwife could help them [40,47].

*“…I don't believe a Somali woman would go and tell her (the midwife) if she is having problems or anything like that…if it has gone far enough that a woman has decided to report the man*, *then she knows she can call the police*, *or that she can get help from friends instead”*. (Byrskog et al 2016, pp. 12) [40]

### CERQual assessment

The summary scores from the CERQual assessment of confidence in the findings can be seen in Table 3 and full details are shown in S4 File. A total of 16 findings were assessed, with twelve scoring high confidence and three scoring moderate confidence and one scoring low confidence.

**Table 3:** CERQual summary scores.

Analytic theme	Review finding	CERQual assessment of confidence in the evidence
Finding the way—Navigating the system in a new place	Migrant women weigh up the value of maternity care and the costs and consequences of accessing care.	**HIGH**
	Some migrant women are unaware of their rights and entitlements to maternity care.	**HIGH**
	Migrant women face difficulties in finding the way into the maternity care system.	**HIGH**
	Ongoing access to maternity care is influenced by financial factors	**HIGH**
	Ongoing access to maternity care is influenced by flexibility in the system	**MODERATE**
We don’t understand each other	Migrant women face language barriers when accessing maternity care	**HIGH**
	Migrant women have unmet perinatal information needs	**MODERATE**
	Migrant women have different expectations of maternity care	**HIGH**
The way you treat me matters	Migrant women experience prejudice and stereotyping from HCPs	**HIGH**
	Maternity care is culturally insensitive to migrant women's needs	**HIGH**
	Migrant women value continuity of care	**MODERATE**
	Migrant women value trusting relationships with HCPs who demonstrate good professional behaviours	**HIGH**
	Migrant women value high quality maternity facilities	**LOW**
My needs go beyond being pregnant	Migrant women face financial difficulties and poor living conditions	**HIGH**
	Migrant women carry the burden of previous traumatic experiences	**HIGH**
	Migrant women have needs related to social support and relationship issues	**HIGH**

## Discussion

### Main findings

Migrant women’s struggles with communication and language barriers are recurrent themes within this and previous reviews. Migrant women report a poor understanding of medical terminology [25] and yet there is inadequate use of interpreters within the healthcare system [25,84]. Poor communication and the provision of insufficient information impact on women’s ability to choose appropriate care options and provide informed consent [25,84–87]. An inability to converse in the local language also means women find it difficult to establish a relationship with their care provider and this impacts upon women accessing care [25,84,88,89]. HCPs can help women to overcome language barriers by providing appropriate information, engaging professional interpreters more frequently and ensuring they give women the opportunity to ask the questions that they have [90–99].

In line with other studies [25,85–87,89,100,101], a lack of understanding between migrants and HCPs in terms of their traditional customs and their expectations of maternity care was found to impact upon their access of services. The issues clearly point to a need for HCPs to receive education and training in culturally competent care to better identify women’s expectations of care and how to understand and appropriately respond to women’s needs related to their cultural background, to ensure effective maternity care and reduce barriers to accessing care [22].

Women’s fear of deportation impacting upon use of services identified within this review is in line with previous literature [88] as is lack of awareness of entitlements to maternity care [86]. The United Nations, to which all European countries belong, has developed the Convention on the Elimination of all Forms of Discrimination Against Women [102] which states that all maternity services, including routine antenatal treatment, must be treated as being immediately necessary; *‘No woman must ever be denied*, *or have delayed*, *maternity services due to charging issues’* (Department of Health and Social Care (2018) p. 67) [103]. Healthcare providers need to ensure the provision of adequate support and timely advice for migrant mothers on their entitlements to care to allay fears and improve access to care, with the ultimate aim of reducing pregnancy complications.

While the healthy migrant phenomenon may mean that some migrants are healthier than the native population [22]; a theme which emerged particularly strongly within this review is that to meet the unique needs of many migrant women there is a necessity for care which goes beyond traditional models. Other academic studies and reports have highlighted migrant women’s unstable or inappropriate living conditions, their financial struggles [25,89,104,105] and the enormous burden of loneliness and the lack of a family network around them [25,85,100,104–106]. As the wider determinants of health are well recognised [107], including intimate partner violence [108], low health literacy [109–111], limited social support [112]; addressing social and mental wellbeing alongside physical wellbeing is seen as important for the overall health of mothers and their infants [113]. Addressing the wider determinants of health which impact on migrant women requires closer cross-agency working with effective collaboration between healthcare, social care, the voluntary sector and communities [2]. This current review also highlighted that many migrant women have experienced trauma prior to and during migration, which is widely recognised to impact on mental health and wellbeing in the destination country [114]. Maternity services should develop trauma-informed care [115] to promote a culture of safety and avoid re-traumatisation through staff training and reviewing policies and procedures through a trauma lens and developing pathways of support to meet the needs of these vulnerable women [115].

Some migrant women described exemplary care, receiving treatment that was empathetic, caring, culturally sensitive and compassionate. However other migrants reported discrimination prevalent in the HCPs that they encountered. Care is seen to be impacted where women do not feel well treated or where they feel discriminated against [84,85], while unrushed, kind, empathetic HCPs are appreciated [25,84,85]. Our findings suggest that continuity of care increases migrant women’s satisfaction with maternity care. This is in line with the Cochrane review into continuity of midwife care models which has found increased satisfaction reported by women receiving continuity by a known midwife, as well as reduced rates of preterm birth and perinatal death [116]. To address the social determinants of health and avoid discriminating against migrant women, it calls for person-centred, high-quality, continuity of care that incorporates aspects of cultural competency and trauma aware care. The evidence within this review, alongside other evidence, led to the development of the ORAMMA integrated perinatal care model [117]. This model has been feasibility tested and will be reported in further articles currently under development. Other known integrated healthcare models include Community Orientated Primary Care [118,119], as well as the integrated approach developed within the European Refugees-Human Movement and Advisory Network (EUR-Human) project [120].

### Strengths and limitations

This review provides up-to-date, systematic evidence located using a comprehensive search undertaken by a multidisciplinary team. Assessing confidence in the evidence using the CERQual approach is a further strength of this review. The review is strengthened by the inclusion of a large number of eligible studies set in 14 different European countries which included migrant women from a wide range of countries of origin. However, some papers did not provide a clear or consistent definition for the term 'migrant' or provide details about how recently the women within their study had arrived in the host country, the specific country of origin or the reason for migration. Hence, some issues that may be more pertinent to particular migrants may not be visible within this synthesis. This review focussed exclusively on migrant women's experiences of maternity care within European host countries. It is recognised that many experiences may overlap with migrant experiences across other world regions for example social isolation, language and cultural barriers. However, to ensure local applicability further in-depth investigation would be required on country or community specific factors influencing migrant experiences.

## Conclusion

There are several implications for practice and research from this review.

- It is important that migrant women feel understood. Professional interpreters should be provided at each appointment/care encounter to enable HCPs to listen to women and build a friendly, trusting relationship with women.
- HCPs should avoid stereotyping and respect and accommodate traditional or cultural practices that are relevant in the perinatal period.
- Migrant women’s needs go beyond their pregnancy and include psychosocial-emotional and economic challenges. To address these needs cross-agency working is needed alongside culturally competent and trauma-informed models of maternity care that incorporates continuity.
- Future research should focus on providing robust evidence on clinical perinatal outcomes for migrant mothers and explore the needs of different migrant populations to facilitate development of tailored interventions.

## Supporting information

S1 File Search strategy. (DOCX)

S2 File Critique tool. (DOCX)

S3 File Excluded studies. (DOCX)

S4 File Full CERQual assessment scoring table. (DOCX)

S1 PRISMA Checklist (DOC)
