# Supplementary material for: Migrant women’s experiences of pregnancy, childbirth and maternity care in European countries: A systematic review
Source: PLoS One. 2020 Feb 11;15(2):e0228378. doi: 10.1371/journal.pone.0228378 (PMC7012401; doi:10.1371/journal.pone.0228378)
Supplement: S1 File — (DOCX) [file pone.0228378.s001.docx]

Supporting Information File 1 - Search strategy

| # | Query |
| --- | --- |
| S1 | migrant |
| S2 | refugee |
| S3 | asylum |
| S4 | immigrant |
| S5 | trafficked |
| S6 | S1 OR S2 OR S3 OR S4 OR S5 |
| S7 | pregnancy |
| S8 | pregnant |
| S9 | maternal |
| S10 | maternity |
| S11 | midwifery |
| S12 | birth |
| S13 | perinatal |
| S14 | intrapartum |
| S15 | antenatal |
| S16 | postnatal |
| S17 | childbearing |
| S18 | prenatal |
| S19 | motherhood |
| S20 | S7 OR S8 OR S9 OR S10 OR S11 OR S12 OR S13 OR S14 OR S15 OR S16 OR S17 OR S18 OR S19 |
| S21 | experience |
| S22 | belief |
| S23 | believe |
| S24 | attitude |
| S25 | view |
| S26 | perspective |
| S27 | story |
| S28 | stories |
| S29 | narrative* |
| S30 | account |
| S31 | accessib* |
| S32 | availab* |
| S33 | acceptab* |
| S34 | quality |
| S35 | help seeking |
| S36 | help-seeking |
| S37 | access |
| S38 | need |
| S39 | S21 OR S22 OR S23 OR S24 OR S25 OR S26 OR S27 OR S28 OR S29 OR S30 OR S31 OR S32 OR S33 OR S34 OR S35 OR S36 OR S37 OR S38 |
| S40 | S6 AND S20 AND S39 |
| S41 | S40 Limiters - Published Date: 20070101-20171231 |
