# Supplementary material for: Migrant women’s experiences of pregnancy, childbirth and maternity care in European countries: A systematic review
Source: PLoS One. 2020 Feb 11;15(2):e0228378. doi: 10.1371/journal.pone.0228378 (PMC7012401; doi:10.1371/journal.pone.0228378)
Supplement: S2 File — (DOCX) [file pone.0228378.s002.docx]

**Supporting Information File 2 - NICE qualitative critique tool** [1]

| Study identification: Include author, title, reference, year of  publication | |  |
| --- | --- | --- |
| **Theoretical** **approach** | | |
| 1. Is a qualitative approach appropriate?  For example:  Does the research question seek to understand processes or structures, or illuminate subjective experiences or meanings?  Could a quantitative approach better have addressed the research question? | Appropriate  Inappropriate  Not sure | **Comments:** |
| 2. Is the study clear in what it seeks to do?  For example:  Is the purpose of the study discussed - aims/objectives/ research question/s?  Is there adequate/appropriate reference to the literature?  Are underpinning values/assumptions/  theory discussed? | Clear  Unclear  Mixed |  |
| **Study design** | | |
| 3. How defensible/rigorous is the research design/ methodology?  For example:  Is the design appropriate to the research question?  Is a rationale given for using a qualitative approach?  Are there clear accounts of the rationale/justification for the sampling, data collection and data analysis techniques used?  Is the selection of cases/sampling strategy theoretically justified? | Defensible  Indefensible  Not sure |  |
| **Data collection** | | |
| 4. How well was the data collection carried out?  For example:  Are the data collection methods clearly described?  Were the appropriate data collected to address the research question?  Was the data collection and record keeping systematic? | Appropriately  Inappropriately  Not sure/  inadequately  reported |  |
| **Trustworthiness** | | |
| 5. Is the role of the researcher clearly described?  For example:  Has the relationship between the researcher and the participants been adequately considered?  Does the paper describe how the research was explained and presented to the participants? | Clearly  described  Unclear  Not described |  |
| 6. Is the context clearly described?  For example:  Are the characteristics of the participants and settings clearly defined?  Were observations made in a sufficient variety of circumstances?  Was context bias considered? | Clear  Unclear  Not sure |  |
| 7. Were the methods reliable?  For example:  Was data collected by more than 1 method?  Is there justification for triangulation, or for not triangulating?  Do the methods investigate what they claim to? | Reliable  Unreliable  Not sure |  |
| **Analysis** | | |
| 8. Is the data analysis sufficiently rigorous?  For example:  Is the procedure explicit – i.e. is it clear how the data was analysed to arrive at the results?  How systematic is the analysis, is the procedure reliable/ dependable?  Is it clear how the themes and concepts were derived from the data? | Rigorous  Not rigorous  Not sure/not  reported |  |
| 9. Is the data 'rich'?  For example:  How well are the contexts of the data described?  Has the diversity of perspective and content been explored?  How well has the detail and depth been demonstrated?  Are responses compared and contrasted across groups/ sites? | Rich  Poor  Not sure/not  Reported |  |
| 10. Is the analysis reliable?  For example:  Did more than 1 researcher theme and code transcripts/ data?  If so, how were differences resolved?  Did participants feedback on the transcripts/data if possible  and relevant?  Were negative/discrepant results addressed or ignored? | Reliable  Unreliable  Not sure/not  reported |  |
| 11. Are the findings convincing?  For example:  Are the findings clearly presented?  Are the findings internally coherent?  Are extracts from the original data included?  Are the data appropriately referenced?  Is the reporting clear and coherent? | Convincing  Not convincing  Not sure |  |
| 12. Are the findings relevant to the aims of the study? | Relevant  Irrelevant  Partially  relevant |  |
| 13. Conclusions  For example:  How clear are the links between data, interpretation and conclusions?  Are the conclusions plausible and coherent?  Have alternative explanations been explored and discounted?  Does this enhance understanding of the research topic?  Are the implications of the research clearly defined?  Is there adequate discussion of any limitations encountered? | Adequate  Inadequate  Not sure |  |
| **Ethics** | | |
| 14. How clear and coherent is the reporting of ethics?  For example:  Have ethical issues been taken into consideration?  Are they adequately discussed e.g. do they address consent and anonymity?  Have the consequences of the research been considered i.e. raising expectations, changing behaviour?  Was the study approved by an ethics committee? | Appropriate  Inappropriate  Not sure/not  reported |  |
| **Overall assessment** | | |
| As far as can be ascertained from the paper, how well was the study conducted? (see guidance notes*) | ++  +  − |  |

Overall scoring

The overall rating of the article was classified as (+) if the answers to questions 1,2,3,11,12,13 were scored highly. Articles were given an overall rating of (++) if questions 5,6,7,8 and 10 also scored highly, as these questions are related to trustworthiness, rigour and reliability. Otherwise articles were classified as (-).

(1) National Institute for Health and Care Excellence. Methods for the development of NICE public health guidance: Process and methods. 2012;Third Edition. NICE(National Institute for Health and Care Excellence): London.
