# Supplementary material for: Migrant women’s experiences of pregnancy, childbirth and maternity care in European countries: A systematic review
Source: PLoS One. 2020 Feb 11;15(2):e0228378. doi: 10.1371/journal.pone.0228378 (PMC7012401; doi:10.1371/journal.pone.0228378)
Supplement: S3 File — (DOCX) [file pone.0228378.s003.docx]

**Supporting Information File 3 – reasons for study exclusion**

| **Excluded from abstract** | | **Excluded from full text** | |
| --- | --- | --- | --- |
| **Study ID** | **Reason for exclusion** | **Study ID** | **Reason for exclusion** |
| Balaam et al (2013) [1] | Review | Akhavan (2012) [39] | Excluded as an intervention study |
| Balaam et al (2016) [2] | Experience of volunteers, not women | Almeida Santos (2014) [40] | No qualitative data reported |
| Benza and Liamputtong (2014) [3] | Review | Banjara (2011) [41] | Thesis, not peer reviewed article |
| Boerleider et al (2013) [4] | Review | Bawadi and Ahmad (2017) [42] | Focus not on experiences of maternity care |
| Bridle (2012) [5] | Review | Berlincionì et al (2014) [43] | Italian article - unable to obtain full text |
| Burström et al (2017) [6] | Disadvantaged women in general - not migrant specific, study protocol | Berlincionì et al (2015) [44] | French article - Unable to retrieve full text |
| Castañeda (2008) [7] | Focus on general healthcare not specifically on perinatal care | Bharji (2007) [45] | Thesis, not peer reviewed article |
| Choté et al (2011) [8] | Outcomes of late antenatal care visits, not women's experiences | Da Lomba and Murray (2014) [46] | Report, not peer reviewed article |
| Dike (2013) [9] | Review | Dayib (2008) [47] | Thesis, not peer reviewed article |
| Dike (2013) [10] | Review | Edge (2008) [48] | Not clear if women are migrants or just from ethnic minority background |
| Douglas (2012) [11] | Not migrant women's experiences | Gaudion (2008) [49] | Report, not peer reviewed article |
| Fisher and Hinchliff (2013) [12] | Review | Gitsels-van der Wal et al (2014) [50] | Role of religion in antenatal screening decision-making, not women's experiences of care |
| Fisher and Hinchliff (2013) [13] | Review | Gitsels-van der Wal et al (2015) [51] | How Moroccan women think about antenatal screening decision-making, not women's experiences of care |
| Goosen et al (2010) [14] | Review | Gonzalez-Pascual et al (2017) [52] | Not experiences of perinatal care |
| Haith-Cooper and McCarthy (2015) [15] | Not migrant women's experiences of perinatal care | Henderson et al (2013) [53] | Women of different ethnic minorities - no description of immigration status |
| Heaman et al (2013) [16] | Review | Higginbottom et al (2013) [54] | Review |
| Hesselink and Harting (2011) [17] | Exclude as an intervention study | Hjelm et al (2009) [55] | Not focussed on experiences of perinatal care |
| Houghton (2008) [18] | Midwife experience, not migrant women's | Hyvönen (2008) [56] | Focus not on experiences of maternity care |
| Jain and Levy (2013) [19] | Review | Keygnaert et al (2016) [57] | Review |
| McCarthy and Haith-Cooper (2013) [20] | Exclude as an intervention study | Lapolla et al (2012) [58] | No qualitative data reported |
| Neale and Wand (2013) [21] | Review | McAree et al (2010) [59] | Women of different ethnic minorities - no description of immigration status |
| Nilaweera et al (2014) [22] | Review | McLeish and Redshaw (2017) [60] | Not migrant women's experiences |
| O'Mahony and Donnelly (2010) [23] | Review | Misfjord (2013) [61] | Thesis, not peer reviewed article |
| O'Mahony et al (2013) [24] | Not migrant women's experiences of maternity care in destination country focus | Moro and Radjack (2012) [62] | Italian article - unable to obtain full text |
| Ostrach (2013) [25] | Review | Özlü and Pruckner (2012) [63] | German article - unable to obtain full text |
| Plaza et al (2014) [26] | Experiences of women of childbearing age, not women who have been pregnant, given birth or postnatal in the destination country | Panaccione and Moro (2014) [64] | Concept of motherhood, not experiences of perinatal care |
| Redshaw and Heikkilä (2011) [27] | Ethnic minority women - no description of immigration status | Paz-Zulueta et al (2015) [65] | Outcome about access to antenatal care - not women's experiences |
| Santiago et al (2015) [28] | Review | Perrin et al (2016) [66] | Case description, not women's experiences of perinatal care |
| Schmied et al (2012) [29] | Review | Philimore and Thornhill (2011) [67] | Report, not peer reviewed article |
| Schmied et al (2017) [30] | Review | Shortall et al (2015) [68] | Report, not peer reviewed article |
| Shandy and Power (2008) [31] | Not migrant women's experiences of maternity care in destination country focus | Waugh (2010) [69] | Report, not peer reviewed article |
| Small et al (2014) [32] | Review |  |  |
| Sudbury and Robinson (2016) [33] | Review |  |  |
| Vasilevski and Carolan-Olah (2016) [34] | Review |  |  |
| Vervliet et al (2014) [35] | Focus on living in Belgium, not on perinatal experiences |  |  |
| Villadsen et al (2016) [36] | Intervention development, not migrant women's experiences |  |  |
| Wikberg and Bondas (2010) [37] | Review |  |  |
| Wittowski et al (2017) [38] | Review |  |  |

**References**

(1) Balaam M, Akerjordet K, Lyberg A, Kaiser B, Schoening E, Fredriksen A, et al. A qualitative review of migrant women's perceptions of their needs and experiences related to pregnancy and childbirth. Journal of Advanced Nursing 2013;69(9):1919-1930.

(2) Balaam M, Kingdon C, Thomson G, Finlayson K, Downe S. 'We make them feel special': The experiences of voluntary sector workers supporting asylum seeking and refugee women during pregnancy and early motherhood. Midwifery 2016;34:133-140.

(3) Benza S, Liamputtong P. Pregnancy, childbirth and motherhood: A meta-synthesis of the lived experiences of immigrant women. Midwifery 2014;30(6):575-584.

(4) Boerleider AW, Wiegers TA, Manniën J, Francke AL, Devillé WLJM. Factors affecting the use of prenatal care by non-western women in industrialized western countries: A systematic review. BMC Pregnancy Childbirth 2013;13:81

(5) Bridle L. Asylum seekers and refugees accessing maternity care-literature review and discussion. Midirs Midwifery Digest 2012;22(1):7-12.

(6) Burström B, Marttila A, Kulane A, Lindberg L, Burström K. Practising proportionate universalism - a study protocol of an extended postnatal home visiting programme in a disadvantaged area in Stockholm, Sweden. BMC Health Services Research 2017;17:1-8.

(7) Castañeda H. Paternity for sale: anxieties over 'demographic theft' and undocumented migrant reproduction in Germany. Medical Anthropology Quarterly 2008;22(4):340-359.

(8) Choté A, Koopmans G, Redekop W, Groot C, Hoefman R, Jaddoe V, et al. Explaining Ethnic Differences in Late Antenatal Care Entry by Predisposing, Enabling and Need Factors in the Netherlands. The Generation R Study. Maternal & Child Health Journal 2011;15(6):689-699.

(9) Dike P. Birth practices of Nigerian women in the UK. British Journal of Midwifery 2013;21(1):41-52.

(10) Dike P. Birth practices of Nigerian women: A literature review. African Journal of Midwifery & Women’s Health 2013;7(1):38-48.

(11) Douglas N. Befriending breastfeeding: a home-based antenatal pilot for South Asian families. Community Practitioner 2012;85(6):28-31.

(12) Fisher J, Hinchliff S. Immigrant women's perceptions of their maternity care: a review of the literature. Part 1. Practising Midwife 2013;16(1):20-22.

(13) Fisher J, Hinchliff S. Immigrant women's perceptions of their maternity care: a review of the literature. Part 2. Practising Midwife 2013;16(2):32-34.

(14) Goosen S, van Oostrum I,E.A., Essink-Bot M. Zwangerschapsuitkomsten en zorgbehoeften bij asielzoeksters (Dutch). [Obstetric outcomes and expressed health needs of pregnant asylum seekers: a literature survey]. Nederlands Tijdschrift voor Geneeskunde 2010;154(47):A2318.

(15) Haith-Cooper M, McCarthy R. Striving for excellence in maternity care: The Maternity Stream of the City of Sanctuary. British Journal of Midwifery 2015;23(9):648-652.

(16) Heaman M, Bayrampour H, Kingston D, Blondel B, Gissler M, Roth C, et al. Migrant Women's Utilization of Prenatal Care: A Systematic Review. Maternal & Child Health Journal 2013;17(5):816-836.

(17) Hesselink AE, Harting J. Process evaluation of a multiple risk factor perinatal programme for a hard-to-reach minority group. Journal of Advanced Nursing 2011;67(9):2026-2037.

(18) Houghton G. Women seeking asylum: are communication needs being met? British Journal of Midwifery 2008;16(3):142-142.

(19) Jain A, Levy D. Conflicting Cultural Perspectives: Meanings and Experiences of Postnatal Depression Among Women in Indian Communities. Health Care for Women International 2013;34(11):966-979.

(20) McCarthy R, Haith-Cooper M. Evaluating the impact of befriending for pregnant asylum-seeking and refugee women. British Journal of Midwifery 2013;21(6):404-409.

(21) Neale A, Wand A. Issues in the evaluation and treatment of anxiety and depression in migrant women in the perinatal period. Australasian Psychiatry 2013;21(4):379-382.

(22) Nilaweera I, Doran F, Fisher J. Prevalence, nature and determinants of postpartum mental health problems among women who have migrated from South Asian to high-income countries: a systematic review of the evidence. Journal of Affective Disorders 2014;166:213-226.

(23) O'Mahony J, Donnelly T. Immigrant and refugee women's post-partum depression help-seeking experiences and access to care: a review and analysis of the literature. Journal of Psychiatric & Mental Health Nursing 2010;17(10):917-928.

(24) O'Mahony JM, Donnelly TT, Raffin Bouchal S, Este D. Cultural background and socioeconomic influence of immigrant and refugee women coping with postpartum depression. Journal of Immigrant and Minority Health 2013;15(2):300-314.

(25) Ostrach B. ' Yo No Sabía...'-Immigrant Women's Use of National Health Systems for Reproductive and Abortion Care. Journal of Immigrant & Minority Health 2013;15(2):262-272.

(26) Plaza SH, Padilla B, Ortiz A, Rodrigues E. The value of grounded theory for disentangling inequalities in maternal-child healthcare in contexts of diversity: A psycho-sociopolitical approach. Psychosocial Intervention 2014;23(2):125-133.

(27) Redshaw M, Heikkilä K. Ethnic differences in women's worries about labour and birth. Ethnicity and Health 2011;16(3):213-223.

(28) Santiago M, Figueiredo M. Immigrant Women's Perspective on Prenatal and Postpartum Care: Systematic Review. Journal of Immigrant & Minority Health 2015;17(1):276-284.

(29) Schmied V, Olley H, Burns E, Duff M, Dennis C, Dahlen HG. Contradictions and conflict: a meta-ethnographic study of migrant women's experiences of breastfeeding in a new country. BMC Pregnancy Childbirth 2012 12/27;12:163-163.

(30) Schmied V, Black E, Naidoo N, Dahlen HG, Liamputtong P. Migrant women's experiences, meanings and ways of dealing with postnatal depression: A meta-ethnographic study. PLoS One 2017;12(3):e0172385.

(31) Shandy DJ, Power DV. The birth of the African-Irish diaspora: Pregnancy and post-natal experiences of African immigrant women in Ireland. International Migration 2008;46(5):119-142.

(32) Small R, Roth C, Raval M, Shafiei T, Korfker D, Heaman M, et al. Immigrant and non-immigrant women's experiences of maternity care: a systematic and comparative review of studies in five countries. BMC Pregnancy Childbirth 2014;14:152

(33) Sudbury H, Robinson A. Barriers to sexual and reproductive health care for refugee and asylum-seeking women. British Journal of Midwifery 2016;24(4):275-281.

(34) Vasilevski V, Carolan-Olah M. Food taboos and nutrition-related pregnancy concerns among Ethiopian women. Journal of Clinical Nursing 2016;25(19):3069-3075.

(35) Vervliet M, De Mol J, Broekaert E, Derluyn I. "That I Live, that's Because of Her": Intersectionality as Framework for Unaccompanied Refugee Mothers. British Journal of Social Work 2014;44(7):2023-2041.

(36) Villadsen SF, Mortensen LH, Andersen AN. Care during pregnancy and childbirth for migrant women: How do we advance? Development of intervention studies--the case of the MAMAACT intervention in Denmark. Best Practice & Research: Clinical Obstetrics & Gynaecology 2016;32:100-112.

(37) Wikberg A, Bondas T. A patient perspective in research on intercultural caring in maternity care: A meta-ethnography. International Journal of Qualitative Studies on Health & Well-being 2010;5(1):1-15.

(38) Wittkowski A, Patel S, Fox JR. The Experience of Postnatal Depression in Immigrant Mothers Living in Western Countries: A Meta-Synthesis. Clinical Psychology & Psychotherapy 2017;24(2):411-427.

(39) Akhavan S, Edge D. Foreign-Born Women's Experiences of Community-Based Doulas in Sweden - A Qualitative Study. Health Care for Women International 2012;33(9):833-848.

(40) Almeida L, Santos CC, Caldas JP, Ayres-de-Campos D, Dias S. Obstetric care in a migrant population with free access to health care. International Journal of Gynaecology & Obstetrics 2014;126(3):244-247.

(41) Banjara TK. Perceptions and experiences of maternity care by South Asian immigrant mothers living in Finland. 2011. Master’s Thesis. Faculty of Health Sciences, University of Eastern Finland: Kuopio.

(42) Bawadi H, Ahmad MM. Childbirth and New Mother Experiences of Arab Migrant Women. MCN: American Journal of Maternal Child Nursing 2017;42(2):101-107.

(43) Berlincionì V, Broglia D, Bruno D, Gambini F, Lalli G, MarelliMarelli C, et al. Diventare madre nella migrazione: Una ricerca qualitativa sulla maternità nel contesto migratorio. (Italian) [Becoming a migrant mother: A qualitative study on motherhood in the context of migration]. Interazioni 2014;1:76-89.

(44) Berlincionì V, Bruno D, Broglia D, Gambini F, Lalli G, Marelli C, et al. Etre mère dans la migration: Quelques réflexions à partir d’une recherche qualitative. (French) [Being a migrant mother: Some reflections from a qualitative study]. La Psychiatrie De L’Enfant 2015;58(2):505-529.

(45) Bharj KK. Pakistani Muslim Women Birthing in Northern England: Exploration of experiences and context. 2007. PhD Thesis. Sheffield Hallam University: Sheffield.

(46) Da Lomba S, Murray N. Women and Children First? Refused asylum seekers’ access to and experiences of maternity care in Glasgow. 2014. Scottish Refugee Council: Glasgow.

(47) Dayib F. The experiences and perception of Somali clients and Finnish nurses in primary health care services. 2008. Master’s Thesis. Department of Nursing Science, University of Kuopio: Kuopio.

(48) Edge D. We don’t see Black women here’: An exploration of the absence of Black Caribbean women from clinical and epidemiological data on perinatal depression in the UK. Midwifery 2008;24(4):379-389.

(49) Gaudion A, Allotey P. Maternity care for asylum seekers and refugees in Hillingdon: A needs assessment (2008) Centre for Public Health Research, Brunel University; Uxbridge.

(50) Gitsels-van der Wal, JT, Manniën J, Ghaly MM, Verhoeven PS, Hutton EK, Reinders HS. The role of religion in decision-making on antenatal screening of congenital anomalies: A qualitative study amongst Muslim Turkish origin immigrants. Midwifery 2014;30(3):297-302.

(51) Gitsels-van der Wal, JT, Martin L, Manniën J, Verhoeven P, Hutton EK, Reinders HS. A qualitative study on how Muslim women of Moroccan descent approach antenatal anomaly screening. Midwifery 2015;31(3):e43-e49.

(52) González-Pascual J,L., Ruiz-López M, Saiz-Navarro EM, Moreno-Preciado M. Exploring Barriers to Breastfeeding Among Chinese Mothers Living in Madrid, Spain. Journal of Immigrant and Minority Health 2017;19(1):74-79.

(53) Henderson J, Gao H, Redshaw M. Experiencing maternity care: the care received and perceptions of women from different ethnic groups. BMC Pregnancy Childbirth 2013;13:196.

(54) Higginbottom G, Reime B, Bharj K, Chowbey P, Ertan K, Foster-Boucher C, et al. Migration and Maternity: Insights of Context, Health Policy, and Research Evidence on Experiences and Outcomes from a Three Country Preliminary Study Across Germany, Canada, and the United Kingdom. Health Care for Women International 2013;34(11):936-965.

(55) Hjelm K, Bard K, Berntorp K, Apelqvist J. Beliefs about health and illness postpartum in women born in Sweden and the Middle East. Midwifery 2009;25(5):564-575.

(56) Hyvönen H. Maa muuttuu, muuttuuko äitiys? Suomalaisten ja virolaisten kokemuksia äitiydestä maahanmuuton jälkeen (Finnish) [The country is changing, does motherhood change? Experiences of motherhood among Finnish and Estonian after immigration]. Yhteiskuntapolitiikka [Politics of the Society] 2008;73(5):508-523.

(57) Keygnaert I, Ivanova O, Guieu A, Van Parys A, Leye E, Roelens K. What is the Evidence on the Reduction of Inequalities in Accessibility and Quality of Maternal Health Care Delivery for Migrants? A Review of the Existing Evidence in the WHO European Region. 2016. WHO Regional Office for Europe: Copenhagen.

(58) Lapolla A, Di Cianni G, Di Benedetto A, Franzetti I, Napoli A, Sciacca L, et al. Quality of Life, Wishes, and Needs in Women with Gestational Diabetes: Italian DAWN Pregnancy Study. International Journal of Endocrinology 2012;784726.

(59) McAree T, McCourt C, Beake S. Perceptions of group practice midwifery from women living in an ethnically diverse setting. Evidence Based Midwifery 2010;8(3):91-97.

(60) McLeish J, Redshaw M. Mothers' accounts of the impact on emotional wellbeing of organised peer support in pregnancy and early parenthood: a qualitative study. BMC Pregnancy Childbirth 2017;17:28.

(61) Misfjord K. The meeting between the midwife and the Somali woman—focusing on health guidance. 2013. Master’s Thesis. Buskerud and Vestfold University College: Horten, Norway.

(62) Moro MR, Radjack R. Approccio transculturale alia perinatalità: Teoria e pratica. (Italian) [Transcultural approach to perinatality: theory and practice]. Infanzia e Adolescenza 2012;11(1):3-10.

(63) Özlü Z, Pruckner F. Migration und Geburtshilfe: Erfahrungen aus einer Spezialambulanz für psychosoziale Krisen in Wien. (German) [Migration and Obstetrics: Experiences form a special outpatient clinic for psychosocial crises in Vienna]. Trauma & Gewalt 2012;6(4):298-305.

(64) Panaccione É, Moro MR. Construire de la sécurité dans I'errance. Maternité chez des femmes migrantes sans domicile fixe (French) [Build security on the journey. Maternity among homeless migrant women]. La Psychiatrie de l’Enfant 2014;57(2):533-561.

(65) Paz-Zulueta M, Llorca J, Santibáñez M. Disparities in Access to Prenatal Care Services for African Immigrant Women in Spain. Journal of Immigrant & Minority Health 2015;17(5):1355-1363.

(66) Perrin A-, Drain E, Sarot A, Moro M-. Comment soutenir l’arrivée au monde d’un enfant de mère migrante dans une maternité Française: Entre urgence somatique et urgence psychiatrique, le temps de la reconstruction…. (French) [How to support the arrival to the world of a child of a migrant mother in a French maternity hospital: Between somatic emergency and psychiatric emergency, the time of reconstruction . . ] Neuropsychiatrie de l’Enfance et de l’Adolescence 2016;64(1):31-35.

(67) Phillimore J, Thornhill J. Delivering in the Age of Superdiversity: West Midlands Review of Maternity Services for Migrant Women. 2011. West Midlands Strategic Migration Partnership and Department of Health: Birmingham.

(68) Shortall C, McMorran J, Taylor K, Traianou A, Garcia de Frutos M, Jones L, et al. Experiences of Pregnant Migrant Women receiving Ante/Peri and Postnatal care in the UK: A Longitudinal Follow-up Study of Doctors of the World’s London Drop-In Clinic Attendees. 2015. Doctors of the World: London.

(69) Waugh M. The Mothers in Exile Project: Women asylum seekers’ and refugees’ experiences of pregnancy and childbirth in Leeds. 2010. Women’s Health Matters: Leeds.
