# Supplementary material for: Migrant women’s experiences of pregnancy, childbirth and maternity care in European countries: A systematic review
Source: PLoS One. 2020 Feb 11;15(2):e0228378. doi: 10.1371/journal.pone.0228378 (PMC7012401; doi:10.1371/journal.pone.0228378)
Supplement: S4 File — (DOCX) [file pone.0228378.s004.docx]

**Supporting Information File 4 - Full CERQual assessment scoring table**

| **Analytic theme** | **Review finding** | **Studies contributing to the review finding** | **Methodological limitations** | **Coherence** | **Adequacy** | **Relevance** | **CERQual assessment of confidence in the evidence** |
| --- | --- | --- | --- | --- | --- | --- | --- |
| Finding the way - Navigating the system in a new place | Migrant women weigh up the value of maternity care and the costs and consequences of accessing care. | [32-36,46,48,49,57,58,62,63,72,78,79] | Moderate methodological limitations  (6 studies low quality, 6 studies moderate quality and 2 studies high quality) | Minor concerns about coherence | Minor concerns about adequacy | No or very minor concerns about relevance | **HIGH** |
|  | Some migrant women are unaware of their rights and entitlements to maternity care. | [31,33,39,48,50,58,62,69,75,79,80] | Moderate methodological limitations  (5 studies low quality, 4 studies moderate quality and 1 study high quality) | Minor concerns about coherence | Minor concerns about adequacy | No or very minor concerns about relevance | **HIGH** |
|  | Migrant women face difficulties in finding the way into the maternity care system. | [32,33,50,58,63,72,77,79] | Minor methodological limitations  (2 studies low quality, 4 studies moderate quality and 1 study high quality) | Minor concerns about coherence | Minor concerns about adequacy | No or very minor concerns about relevance | **HIGH** |
|  | Ongoing access to maternity care is influenced by financial factors | [31,34,41,46,50,58,64,78,80] | Minor methodological limitations  (4 studies low quality, 3 studies moderate quality and 2 studies high quality) | Minor concerns about coherence | Minor concerns about adequacy | No or minor concerns about relevance | **HIGH** |
|  | Ongoing access to maternity care is influenced by flexibility in the system | [31,58,62,67,72,79] | Minor methodological concerns  (1 study low quality, 4 studies moderate quality, 1 study high quality) | Minor concerns about coherence | Moderate concerns about adequacy | No or minor concerns about relevance | **MODERATE** |
| We don’t understand each other | Migrant women face language barriers when accessing maternity care | [32,34,35,41,44,48-50,55,57,63,66,67,69,71,72,75,78-80] | Minor methodological concerns  (9 studies low quality, 5 studies moderate quality and 3 studies high quality) | Minor concerns about coherence | No or very minor concerns about adequacy - rich data overall from multiple sources | No or very minor concerns about relevance | **HIGH** |
|  | Migrant women have unmet perinatal information needs | [8,32,34,38,43-47,49,51,55,60,61,63,67-69,72-76,78-80] | Minor methodological concerns  (10 studies low quality, 10 studies moderate quality and 3 studies high quality) | Moderate concerns about coherence | No or very minor concerns about adequacy - rich data overall from multiple sources | No or very minor concerns about relevance | **MODERATE** |
|  | Migrant women have different expectations of maternity care | [8,36,39,40,46,52,53,57,58,60,64,67,68,71,77-79] | Minor methodological concerns  (3 studies low quality, 9 studies moderate quality and 5 studies high quality) | Minor concerns about coherence | No or very minor concerns about adequacy - rich data overall from multiple sources | No or very minor concerns about relevance | **HIGH** |
| The way you treat me matters | Migrant women experience prejudice and stereotyping from HCPs | [8,32,34,47,49,50,54,55,63,66,72-75] | Moderate methodological limitations  (7 studies low quality, 6 studies moderate quality, 1 study high quality) | Minor concerns about coherence | Minor concerns about adequacy | No or very minor concerns about relevance | **HIGH** |
|  | Maternity care is culturally insensitive to migrant women's needs | [8,32,34,42,49-52,54,55,61,63,64,66,72-75,80] | Minor methodological limitations  (7 studies low quality, 8 studies moderate quality, 3 studies high quality) | Minor concerns about coherence | Minor concerns about adequacy | No or very minor concerns about relevance | **HIGH** |
|  | Migrant women value continuity of care | [48,55,60,72,75,78,79] | Minor methodological limitations  (2 studies low quality, 3 studies moderate quality, 1 study high quality) | Minor concerns about coherence | Moderate concerns about adequacy | No or very minor concerns about relevance | **MODERATE** |
|  | Migrant women value trusting relationships with HCPs who demonstrate good professional behaviours | [34,40,43,45-47,49,54,57-60,63,64,67,68,71,72,74,75,77-79] | Minor methodological limitations  (5 studies low quality, 12 studies moderate quality, 5 studies high quality) | Minor concerns about coherence | No or very minor concerns about adequacy - rich data overall from multiple sources | No or very minor concerns about relevance | **HIGH** |
|  | Migrant women value high quality maternity facilities | [34,71,75,78] | Minor methodological limitations  (1 study low quality, 1 study moderate quality, 1 study high quality) | Minor concerns about coherence | Serious concerns about adequacy | Moderate concerns about adequacy | **LOW** |
| My needs go beyond being pregnant | Migrant women face financial difficulties and poor living conditions | [32,33,41,46,48-50,58-60,62,63,67,69-72,78-80] | Minor methodological concerns  (7 studies low quality, 9 studies moderate quality and 2 studies high quality) | Minor concerns about coherence | Minor concerns about adequacy - rich data overall from multiple sources | No or very minor concerns about relevance | **HIGH** |
|  | Migrant women carry the burden of previous traumatic experiences | [42,49,55,57-60,67,72] | Minor methodological concerns  (2 studies low quality, 6 studies moderate quality and 1 study high quality) | Minor concerns about coherence | No or very minor concerns about adequacy - rich data overall from multiple sources | No or very minor concerns about relevance | **HIGH** |
|  | Migrant women have needs related to social support and relationship issues | [32,34,37,41,42,44,46,48,50,53,55-58,60,61,65,67,68,70-72,74-76,78,80] | Minor methodological concerns  (8 studies low quality, 11 studies moderate quality and 6 studies high quality) | Minor concerns about coherence | No or very minor concerns about adequacy - rich data overall from multiple sources | No or very minor concerns about relevance | **HIGH** |
